# Dietary fat intake is associated with insulin resistance and an adverse vascular profile in patients with T1D: a pooled analysis

**DOI:** 10.1007/s00394-022-03070-z

**Published:** 2022-12-10

**Authors:** Noppadol Kietsiriroje, Hanya Shah, Marios Zare, Lauren L. O’Mahoney, Daniel J. West, Sam M. Pearson, Ramzi A. Ajjan, Matthew D. Campbell

**Affiliations:** 1 Leeds Institute of Cardiovascular and Metabolic Medicine, Faculty of Medicine and Health, University of Leeds, Leeds, UK; 2 Endocrinology and Metabolism Unit, Faculty of Medicine, Prince of Songkla University, Songkhla, Thailand; 3 Faculty of Health Sciences and Wellbeing, University of Sunderland, Sunderland, SR1 3SD UK; 4 Diabetes Research Centre, Leicester General Hospital, University of Leicester, Leicester, UK; 5 Human Nutrition Research Centre, Population Health Sciences Institute, Faculty of Medical Sciences, Newcastle University, Newcastle, UK; 6 Insutiv, T1D One Life Ltd, London, UK

**Keywords:** Type 1 diabetes, Dietary intake, Insulin resistance, Vascular health

## Abstract

**Background:**

Insulin resistance (IR) increases vascular risk in individuals with Type 1 Diabetes (T1D). We aimed to investigate the relationship between dietary intake and IR, as well as vascular biomarkers in T1D.

**Methods:**

Baseline data from three randomised controlled trials were pooled. Estimated glucose disposal rate (eGDR) was used as an IR marker. Employing multivariate nutrient density substitution models, we examined the association between macronutrient composition and IR/vascular biomarkers (tumour necrosis factor-α, fibrinogen, tissue factor activity, and plasminogen activator inhibitor-1).

**Results:**

Of the 107 patients, 50.5% were male with mean age of 29 ± 6 years. Those with lower eGDR were older with a longer diabetes duration, higher insulin requirements, and an adverse vascular profile (*p* < 0.05). Patients with higher degrees of IR had higher total energy intake (3192 ± 566 vs. 2772 ± 268 vs. 2626 ± 395 kcal/d for eGDR < 5.1 vs. 5.1–8.6 vs. ≥ 8.7 mg/kg/min, *p* < 0.001) and consumed a higher absolute and proportional amount of fat (47.6 ± 18.6 vs. 30.4 ± 8.1 vs. 25.8 ± 10.4%, *p* < 0.001). After adjusting for total energy intake, age, sex, and diabetes duration, increased carbohydrate intake offset by an isoenergetic decrease in fat was associated with higher eGDR (*β* = 0.103, 95% CI 0.044–0.163). In contrast, increased dietary fat at the expense of dietary protein intake was associated with lower eGDR (*β* = − 0.119, 95% CI − 0.199 to − 0.040). Replacing fat with 5% isoenergetic amount of carbohydrate resulted in decreased vascular biomarkers (*p* < 0.05).

**Conclusion:**

Higher fat, but not carbohydrate, intake is associated with increased IR and an adverse vascular profile in patients with T1D.

## Introduction

Weight gain and insulin resistance (IR) in type 1 diabetes (T1D) are prevalent and a significant source of morbidity and mortality [1]. In the most recent UK National Diabetes Audit, 63.5% of individuals with T1D were classified as overweight or obese [2]—a phenotype which expresses greater IR and a concomitant increased risk of vascular complications irrespective of glycaemic control [1, 3]. Mechanisms contributing to the increased risk of vascular complications partly due to interaction between insulin resistance and inflammation creating prothrombotic environment [4]. Elevation of vascular biomarkers, such as tumour necrotic factor-alpha (TNF-α), fibrinogen, tissue factor (TF) activity, and plasminogen activator inhibitor-1 (PAI-1) [5], has been linked to endothelial dysfunction, prothrombotic clot formation and hypofibrinolysis, thus resulting in increased vascular risk for atherosclerosis [5, 6].

Excessive energy intake leads to weight gain and predisposes to IR. However, it is widely acknowledged that individual dietary macronutrients consumed in differing isoenergetic quantities have differential effects on IR and vascular risk factors [7]. Recently, it has been shown that in T1D, the relative distribution of dietary macronutrients was associated with the presence of the metabolic syndrome components [8], in men but not women. Favouring carbohydrate intake over fat was associated with lower waist circumference and that favouring either carbohydrates or fat over protein was associated with a lower prevalence of blood pressure [8]. To the best of our knowledge, the association between macronutrient intake and IR—a key mechanistic driver of increased vascular complications—has never been evaluated in people with T1D.

In T1D, the focus on carbohydrate intake is often emphasised whereby structured education provided to patients for managing mealtime insulin dose is centred on total carbohydrate amount [9]. However, preference for high fat and protein over carbohydrate has been previously reported [10] and anecdotally, within the T1D community, there is often a concern that increased carbohydrate intake increases IR and worsens glucose management and that carbohydrate restriction should be promoted. In the present study, we pooled pre-treatment data from three randomised controlled trials (RCTs) and employed a multivariable nutrient density substitution model to assess the association between relative macronutrient components with IR and vascular biomarkers in a population of well-defined T1D patients.

## Methods

### Study population

We pooled data from three randomised controlled trials (RCTs; Clinical trial registration: ISRCTN40811115; ISRCTN13641847, NCT05231642) each of which received ethical approval from local National Health Service Research Ethics Committees (REC reference: 17/NE/0244, 20/LO/0650, 21/WA/0381.) Written informed consent was obtained from all participants. In the present analysis, we included 107 participants that met inclusion criteria as described previously [11, 12] including classical presentation of T1D, aged 18–50 years, diabetes duration of ≥ 5 years, treated on a stable (> 12-months) basal-bolus insulin regimen delivered through multiple daily injections or continuous subcutaneous insulin infusion and no established diabetes-related complications.

### Data collection and study procedures

We performed cross-sectional-analyses using baseline pre-treatment data across each RCT. Overnight fasting venous blood samples were obtained and analysed for plasma levels of vascular biomarkers, including TNF-α (Human TNF-α Quantikine ELISA; R&D Systems, Roche Diagnostics, UK), fibrinogen (ab108842, Fibrinogen Human ELISA Kit; Abcam, Japan), TF activity (Human Tissue Factor activity ab108906; Abcam, UK), and PAI-1 activity (Human PAI-1/serpin ELISA Kit DSE100; R&D systems, UK). Estimated glucose disposal rate (eGDR) was calculated using a validated formula: eGDR = 19.02−(0.22 × BMI [kg/m^2^])−(3.26 × HTN)−(0.61 × HbA1c [%]), whereby HTN is hypertension (1 = yes, 0 = no) [4]. Participants were defined as hypertensive if blood pressure ≥ 140/90 mmHg, they had a pre-existing diagnosis of hypertension or were prescribed antihypertensive drugs.

To estimate dietary intake, participants completed two independent dietary assessments; a 48-h weighed food diary and a validated DINE Food Frequency Questionnaire (FFQ) [13]. We employed both assessment techniques to facilitate cross-validation and improve accuracy of reporting [14]. Analysis of the 48-h weighed food diary was performed using the validated MyFood24 tool [15]. Using the DINE method, fat intake [13] was categorised into three groups whereby frequencies of fat consumption reported by patients were translated into a score. A DINE fat score < 30 (equivalent to ≤ 83 g/day) indicates low fat intake (DINE1), whereas 30–40 (equivalent to 84–122 g/day) and score > 40 (equivalent to > 122 g/day) indicate moderate (DINE2) and high-fat intake (DINE3), respectively.

### Statistical analysis

Baseline characteristics were presented according to eGDR tertiles. Continuous variables are reported as mean ± SD and categorical variables are reported as frequency (percentage). Conditional differences assessed used one-way ANOVA with post hoc Bonferroni for continuous variables and Chi-square for categorical variables. Associations between macronutrient intake and IR were investigated using a generalised linear regression analysis whereby eGDR was entered as a dependent variable and one macronutrient (from the 48-h weighed food diary) at a time was entered as an independent variable with total energy intake, age, sex, and diabetes duration used as covariates.

We employed a series of multivariate nutrient density substitution models to examine the effect of increasing an isoenergetic amount of one macronutrient at the expense of another on IR and vascular biomarkers. This technique has been described in detail elsewhere [8] but in brief, a series of sequential generalised linear regression analyses were performed featuring either eGDR or vascular biomarkers as a dependent variable; Using macronutrient variables assessed by the 48-h weighed food diary, we included all but one macronutrient (per 5 E% presented in parentheses) and total energy intake as covariates; in a second adjusted model, we included age, sex, and diabetes duration as additional covariates. For example, in a model replacing fat with a 5% isoenergetic amount of carbohydrate [↑CHO (↓FAT)], the %E of carbohydrate after 5% isoenergetic substitution was entered as an independent variable, whereas fat was excluded from the model. Protein intake, total energy intake, and other selected variables were used as covariates. The results can be interpreted as the increase or decrease in the dependent outcome related to isoenergetic (5E%) substitution of a given macronutrient in the model with the macronutrient omitted from the model. For instance, in an equation: eGDR = β0 + β1 (5E% increase from carbohydrates) + β2 (E% from protein) + β3 (total energy intake), β1 would be interpreted as the change in the eGDR value when dietary carbohydrate intake is increased by 5E% at the expense of fat.

Data were analysed using SPSS (IBM SPSS Statistics 25, IBM Corporation, USA). Statistical significance was set at *p* < 0.05 for all analyses.

## Results

Our study population consisted of *n* = 107 patients with T1D. We stratified this cohort by eGDR tertiles, with lower eGDR values conferring higher degrees of IR. Baseline demographic and clinical characteristics are presented. Of the 107 patients, 50.5% were male with mean age of 29 ± 6 years. Those with lower eGDR were typically older, (Table 1) had a longer diabetes duration, required higher insulin doses, and had an adverse vascular profile (*p* < 0.05).

**Table 1:** Clinical characteristics, vascular biomarker levels, and nutritional intake of the study population categorised by eGDR tertiles (*n* = 107)

	All patients	eGDR tertiles (mg/kg/min)			*p* value
		< 5.1	5.1–8.6	≥ 8.7	
*N*	107	36	38	33	
Clinical characteristics					
Sex (%male)	50.5%	52.8%	50.0%	48.5%	0.936
Age (years)	29 ± 6	32 ± 6	28 ± 5*	27 ± 5*	< 0.001
BMI	26.3 ± 3.4	28.7 ± 3.5	26.6 ± 2.4*	23.3 ± 1.4*	< 0.001
Hypertension (%)	44.9%	100%	31.6%	0%	< 0.001
HbA1c (%) [mmol/mol]	8.0 ± 1.2 [63.8 ± 12.8]	9.0 ± 1.0 [75.1 ± 11.4]	7.8 ± 1.0* [62.3 ± 10.9]*	7.0 ± 0.3* [53.4 ± 2.8]*	< 0.001
eGDR (mg/kg/min)	6.9 ± 2.5	3.9 ± 1.0	7.3 ± 1.2*	9.6 ± 0.4*	< 0.001
Diabetes duration (years)	16.5 ± 7.0	19.5 ± 8.4	17.6 ± 4.3	11.9 ± 5.5*	< 0.001
Daily insulin dose (units)	47 ± 15	55 ± 20	47 ± 10*	39 ± 5*	< 0.001
Vascular biomarker levels					
TNF-α (pg/mL)	4.23 ± 1.65	5.99 ± 1.31	3.83 ± 0.81*	2.77 ± 0.74*	< 0.001
Fibrinogen (mg/mL)	2.19 ± 1.14	3.38 ± 0.89	1.87 ± 0.69*	1.27 ± 0.54*	< 0.001
TF activity (units/mL)	70.5 ± 30.4	102.4 ± 19.5	63.3 ± 19.6*	44.0 ± 16.3*	< 0.001
PAI-1 (pg/mL)	1274 ± 657	1995 ± 487	1059 ± 366*	735 ± 282*	< 0.001
Nutritional intake from 48-h weighed food diary					
Energy intake (Kcal/d)	2869 ± 485	3192 ± 566	2772 ± 268*	2626 ± 395*	< 0.001
Carbohydrate (% energy)	48.3 ± 17.1	35.8 ± 18.7	53.0 ± 12.2*	56.6 ± 12.0*	< 0.001
Fat (% energy)	34.8 ± 16.1	47.6 ± 18.6	30.4 ± 8.1*	25.8 ± 10.4*	< 0.001
Protein (% energy)	16.9 ± 7.4	16.5 ± 8.8	16.6 ± 6.6	17.6 ± 6.7	0.798
DINE assessment					
* N*	93	35	33	25	
Total fat rating	30.4 ± 13.1	42.9 ± 11.2	27.2 ± 5.4*	17.1 ± 3.8*	< 0.001
Unsaturated fat rating	8.6 ± 2.8	8.4 ± 2.3	8.2 ± 2.8	9.4 ± 3.2	0.272
Fibre rating	29.6 ± 14.3	30.5 ± 16.6	30.8 ± 12.0	26.6 ± 13.8	0.475
DINE fat group (%)					
1 Low fat	54.8%	0%	78.8%	100%	< 0.001
2 Medium fat	26.9%	54.3%	18.2%	0%	
3 High fat	18.3%	45.7%	3.0%	0%	

Continuous variables are reported as mean ± SD; categorical variables are reported as frequency (percentage). Conditional differences assessed used one-way ANOVA for continuous variables and Chi-square for categorical variables

*TNF-α* tumour necrotic factor-alpha, *TF* tissue factor, *PAI-1* plasminogen activator inhibitor-10

*Post hoc Bonferroni *p* < 0.05 compared with 1st tertile

When evaluating self-reported 48-h dietary intake in our patients, daily energy intake was inversely correlated with eGDR, with patients in the lowest eGDR tertile (i.e., highest degree of IR), consuming the greatest energy intakes. When exploring differences in macronutrient intake, patients with lowest eGDR reported higher-fat and lower-carbohydrate intakes. Protein intake was similar across eGDR tertiles. When categorising patients based on the DINE method, a similar pattern was evident, with fat intake increasing in a stepwise fashion with decreasing eGDR. Unsaturated fat and fibre levels were similar across eGDR tertiles (Table 2).

**Table 2:** Association between macronutrient intake from the 48-h weighed food diary and eGDR in patients with T1D

	Model 1		Model 2	
	*β* (CI)	*p* value	*β* (CI)	*p* value
Carbohydrate	**0.046 (0.005** to **0.087)**	0.029*	0.031 (**− **0.005 to 0.067)	0.094
Fat	**− 0.129 (− 0.196** to **− 0.062)**	< 0.001**	**− 0.101 (− 0.161** to − **0.042)**	0.001*
Protein	**− **0.002 (**− **0.059 to 0.054)	0.938	0.008 (**− **0.042 to 0.057)	0.764

Model 1 is adjusted for energy intake, and Model 2 was fit to estimate associations with adjustment for age, sex, and diabetes duration and energy intake

*and bold text denotes significant association at *p* < 0.05

**and bold text denotes a significant association at *p* < 0.001

To investigate the relationship between IR and macronutrient intake further, we employed a series of multivariate nutrient density models to examine the associations between dietary macronutrient amounts from the 48-h food diary and IR. The percentage of energy from carbohydrate was positively associated with eGDR (greater insulin sensitivity), and fat inversely associated with eGDR (greater IR) in unadjusted models; the association between fat and eGDR remained robust following adjustment. We then studied the association of the relative proportions of dietary macronutrients with IR in adjusted energy-controlled substitution models. These models indicate that increased carbohydrate intake offset by an isoenergetic decrease in fat is associated with higher eGDR (decreased IR) and that increased dietary fat at the expense of dietary carbohydrate or protein intake is associated with lower eGDR (increased IR) (Table 3)

**Table 3:** The changes in eGDR levels after isoenergetic substitution of one macronutrient (from 48-h weighed food diary) to another (in parenthesis) by 5% of total energy (*n* = 107)

	Model 1		Model 2	
	*β* (CI)	*p* value	*β* (CI)	*p* value
↑CHO (↓Fat)	**0.130 (0.063** to **0.198)**	< 0.001**	**0.103 (0.044** to **0.163)**	0.001*
↑CHO (↓Protein)	− 0.010 (− 0.063 to 0.044)	0.720	− 0.016 (− 0.063 to 0.031)	0.504
↑Fat (↓Protein)	− **0.014 (**− **0.231** to − **0.049)**	0.003*	− **0.119 (**− **0.199** to − **0.040)**	0.003*

Model 1 is adjusted for energy intake; Model 2 is fit to estimate associations with adjustment for age, sex, and diabetes duration and energy intake. In each model, a given macronutrient is included as an independent variable and one of the macronutrients (in parentheses) is excluded from the model. The remaining macronutrients and other covariates (total energy intake, age, sex, and diabetes duration) are included as covariates. The *β* represents the increase or decrease in the eGDR variable when increasing the intake of the independent macronutrient by 5% of total energy, while simultaneously reducing an isoenergetic amount of the excluded macronutrient [8]

*and bold text denotes significant association at *p* < 0.05

**and bold text denotes a significant association at *p* < 0.001

When evaluating the relationship between macronutrients and vascular biomarkers, substituting fat with a 5% isoenergetic amount of carbohydrate resulted in a decrease across all chosen vascular biomarkers in unadjusted analyses. Following adjustment for age, sex, and diabetes duration, the associations remained robust for PAI-1, (Table 4) TF activity, and Fibrinogen, but not TNF-α. Conversely, substituting protein with fat resulted in a concomitant increase in biomarkers in unadjusted analyses, but the association was no longer significant for TNF-α and TF activity following adjustment.

**Table 4:** The changes in vascular biomarker levels after isoenergetic substitution of one macronutrient from 48-h weighed food diary to another (in parenthesis) by 5% of total energy (*n* = 107)

	TNF-α (pg/mL)		Fibrinogen (mg/mL)		TF activity (units/mL)		PAI-1 (pg/mL)	
	*β* (95% CI)	*p* value	*β* (95% CI)	*p* value	*β* (95% CI)	*p* value	*β* (95% CI)	*p* value
Model 1								
↑CHO (↓Fat)	**− 0.055 (− 0.101, − 0.009)**	0.020*	**− 0.041 (− 0.073, − 0.009)**	0.012*	**− 1.408 (− 2.253, − 0.562)**	0.001**	**− 31.22 (− 49.39, − 13.05)**	0.001*
↑CHO (↓Protein)	0.011 (**− **0.025, 0.048)	0.551	0.007 (**− **0.018, 0.033)	0.576	**− **0.173 (**− **0.843, 0.497)	0.613	0.415 (**− **13.99, 14.82)	0.955
↑Fat (↓Protein)	**0.066 (0.004, 0.128)**	0.037*	**0.048 (0.005, 0.091)**	0.028*	**1.235 (0.097, 2.373)**	**0.033**	**31.64 (7.18, 56.11)**	0.011*
Model 2								
↑CHO (↓Fat)	**− **0.037 (**− **0.077, 0.002)	0.064	**− 0.029 (− 0.058, − 0.001)**	0.042*	**− 1.064 (− 1.804, − 0.324)**	0.005**	**− 24.75 (− 40.91, − 8.60)**	0.003*
↑CHO (↓Protein)	0.013 (**− **0.018, 0.045)	0.398	0.009 (**− **0.013, 0.032)	0.408	**− **0.082 (**− **0.664, 0.501)	0.783	1.47 (**− **11.25, 14.19)	0.821
↑Fat (↓Protein)	0.051 (**− **0.002, 0.104)	0.059	**0.039 (0.001, 0.077)**	0.045*	0.982 (**− **0.005, 1.970)	0.051	**26.22 (4.65, 47.80)**	0.017*

Model 1 is adjusted for energy intake; Model 2 is Model 1 with further adjustment for age, sex, and diabetes duration and energy intake. In each model, a given macronutrient is included as an independent variable and one of the macronutrients (in parentheses) is excluded from the model. The remaining macronutrient and other confounders (total energy, age, sex, and diabetes duration) are included as covariates. The *β* represents the increase or decrease in the vascular biomarkers when increasing the intake of the independent macronutrient by 5% of total energy, while simultaneously reducing an isoenergetic amount of the excluded macronutrient [8]

*and bold text denotes significant association at *p* < 0.05

** and bold text denotes a significant association at *p* < 0.001

## Discussion

To the best of our knowledge, this is the first report showing that dietary intake is associated with IR and an adverse vascular profile in patients with T1D. Patients with a high degree of IR tended to have higher overall energy intakes resulting largely from a relatively higher-fat intake, as compared to those with lower degrees of IR. Utilizing our nutrient substitutional model, we reveal that increasing amounts of carbohydrate offset by an isoenergetic decrease in fat results in greater insulin sensitivity levels, whereas increased dietary fat at the expense of dietary carbohydrate or protein intake is associated with increased IR. We also show that increased fat consumption is associated with an adverse vascular biomarker profile, whereas replacing fat intake with carbohydrate is associated with a favourable vascular biomarker profile.

The link between IR (assessed by eGDR) and vascular health has been demonstrated recently by our group [1, 16] and others [17, 18], whereby IR increases risk of vascular complications in T1D. Increased total energy intake was associated with IR, which, in this study, was mainly driven by increased fat consumption. In our patients categorised in the lowest eGDR tertile, energy from fat accounted for ~ 48% of total energy intake, as compared to ~ 26–30% in other eGDR tertiles. Importantly, the association between fat intake and IR remained robust in our nutrient substitution model whereby one macronutrient is substituted for an isoenergetic amount of another. Given the cross-sectional nature of the present study with self-report diet data captured at a single time-point, conclusions should be interpreted with caution. However, a plausible explanation for our findings is that a higher proportion of fat at a given energy intake, on average, induces IR to a greater degree than an equivalent calorific amount of carbohydrate, thus favouring a lower-fat and higher-carbohydrate diet. Data from short-term preliminary clinical studies are equivocal with regards to the metabolic advantages of lower-fat higher-carbohydrate verses lower-carbohydrate higher-fat diets [19]. However, in animal models, high-fat high calorie feeding has been shown to dramatically induce IR [20, 21], and in our acute feeding studies in humans with T1D, we have shown that a high-fat feeding challenge promotes adverse glucose and inflammatory profiles and increases insulin requirements [11].

In T1D, preference for high fat and protein over carbohydrate has been previously reported [10]. Within the T1D community, there is often a concern that increased carbohydrate intake increases IR and worsens glucose management and that carbohydrate intake should be restricted [22]. However, our cross-sectional data do not support this notion, whereby increased carbohydrate intake was associated with lower IR, improved glucose management (HbA1c), and a more favourable vascular profile. These findings, albeit preliminary, support previously published research in which high-fat intake was associated with increased coronary heart disease risk and coronary artery calcium in a cohort of 571 individuals with T1D [10].

In general, most dietary guidelines focus predominately on single nutrients, recommending to reduce saturated fat consumption and advocating whole foods over those foods which are heavily processed [23–25]. Beyond macronutrients, diet quality and food processing are important considerations. For example, we have previously shown that T1D individuals express differential fatty acid profiles with regards to IR status and vascular biomarkers [26] and that postprandial vascular-inflammatory and thrombotic responses to high-fat feeding are influenced differentially not only be total fat amount, but also food processing [27].

The impact of high-fat intake (particularly saturated fatty acids, SFAs) on IR is heavily mediated by inflammatory processes [28] directly inducing multiple pleiotropic proinflammatory pathways. Namely, activation of Toll-like receptor-4 pathway which further activates secondary cascades such as c-Jun N-terminal kinase, nuclear factor-kappa B, and protein C kinase signalling pathways which are implicated in the desensitisation of insulin receptors.[28] A cross-sectional analysis in 555 patients with T2D from the Insulin Resistance Atherosclerosis Study has also demonstrated associations between serum total SFA and various vascular-inflammatory markers, including PAI-1, TNF-α, and fibrinogen [29]. In the present study, those individuals with the greatest fat intake also consumed the largest absolute and relative amounts of saturated fat. Therefore, we cannot exclude the possibility that the associations between fat intake and IR/vascular biomarkers were driven not only be fat amount, but also fat type. Due to data structure and sample size, it was not possible to test the hypothesis that substituting an isoenergetic amount of SFA’s at the expense of unsaturated fat increases IR and worsens vascular biomarkers. However, an extensive review of studies in non-T1D individuals shows that substitution of SFAs by isocaloric exchange with monosaturated fatty acids (MUFAs) or polysaturated fatty acids (PUFAs) improves lipid metabolism (including lower levels of LDL-C, triglycerides, ApoB, and ApoA-I, as well as total cholesterol:HDL-C ratio), and glucose homeostasis (including lower HbA1c and IR measured as HOMA-IR), although findings were less conclusive regarding the impact of this on cardiovascular disease risk [30]. Further, results from the OmniHeart trial demonstrated that replacing carbohydrate with unsaturated but not saturated fat improves insulin sensitivity in individuals with pre-hypertension or hypertension stage I without diabetes [31]. A meta-analysis has shown that substituting carbohydrate or PUFA-enriched diets with an MUFA-enriched diet, improved body weight, fasting glucose, lipid profiles, and blood pressure in 1,460 people with T2D [32].

While our pooled retrospective analysis is the first to explore and offers valuable insight into the association between dietary fat intake with IR and vascular health, this study is not without limitations and include (1) our cross-sectional design featuring self-reported dietary intake at a single time-point. Whereas it is possible that increasing fat intake may increase IR, it is also possible that those patients presenting with IR may have previously transitioned to a lower-carbohydrate diet. (2) Self-report dietary assessments have inherent limitations, although our results were consistent between the self-reported weighed food diary and validated FFQ. We used a weighted food diary to obtain accurate estimates of food intake; however, there are known limitations of this including increased participant burden, participant biases, and issues regarding the representative nature of acute versus longer-term dietary patterns. Therefore, we also employed a brief (to minimise participant burden) DINE FFQ which captures generalised long-term dietary patterns. (3) From our current analysis, it was not possible to assess diet quality and food processing, or individual nutrient sub-groups which have previously been shown to impact metabolic health [27, 33] beyond dietary macronutrient distribution. (4) The association of dietary components with outcomes of interest are likely to be non-linear [34]. To address these limitations, a longitudinal observation in a larger representative sample assessing diet in more detail, specifically the threshold at which dietary components increase risk of IR, is warranted.

## Conclusion and future direction

This is the first study to demonstrate dietary macronutrient intake, specifically higher-fat intake, is associated with IR and an adverse vascular profile in patients with T1D. Patients with higher degrees of IR presented with higher total energy intakes and consumed a higher absolute and proportional amount of dietary fat. In the present study, patients with IR and high dietary fat intakes presented with an adverse vascular profile. Future research is required to explore the impact of diet in greater detail with a specific focus on individual dietary components, including diet quality, processing, and timing, thus enabling more accurate and personalized individually dietary management. A different dietary assessment tool such as 7-day food diary may also be required to better display dietary patterns in this group.

## Data Availability

The datasets generated during and/or analysed during the current study are available from the corresponding author on reasonable request.
